# Effortful Processing Reduces the Attraction Effect in Multi-Alternative Decision Making: An Electrophysiological Study Using a Task-Irrelevant Probe Technique

**DOI:** 10.3389/fpsyg.2019.00896

**Published:** 2019-04-24

**Authors:** Takashi Tsuzuki, Yuji Takeda, Itsuki Chiba

**Affiliations:** ^1^Department of Psychology, College of Contemporary Psychology, Rikkyo University, Niiza, Japan; ^2^National Institute of Advanced Industrial Science and Technology, Tsukuba, Japan

**Keywords:** decision making, attraction effect, cognitive resource, task-irrelevant probe, event-related potential

## Abstract

The attraction effect in multi-alternative decision making reflects the context-dependent violation of axioms that are considered fundamental to rational choice. This effect is believed to depend on relatively effortless and intuitive processing (System 1) rather than on effortful and elaborative processing (System 2). To investigate the relationship between cognitive resources and the attraction effect in detail, we used a task-irrelevant probe technique, wherein task-irrelevant auditory probes were presented while participants viewed each alternative in a decision-making task, and measured the electroencephalographic responses to the probes. Thirty participants solved 48 hypothetical purchase problems with three alternatives that differed in terms of two attributes. We found that, in the second epoch of the experimental trials (possibly corresponding to the evaluation and comparison stage), the mean N1 amplitudes of the event-related potentials elicited by the auditory probes were significantly smaller when participants chose the competitor (i.e., trials in which no attraction effect occurred) than when participants chose the target (i.e., trials in which an attraction effect may have occurred). This result suggests that the allocation of more cognitive resources to the alternatives disrupts the attraction effect. This finding supports the assumption that intuitive comparisons among alternatives executed by System 1 are critical for the occurrence of the attraction effect.

## Introduction

Rational theories of decision making have suggested that choice is intrinsically determined by the utilities of the individual alternatives and thus unaffected by the relationships among the alternatives that are part of the choice context. However, many recent studies have described violations of this tenet. In multi-alternative decision making, the attraction effect is considered a form of irrational choice, because it violates the regularity principle of rational choice (Roe et al., 2001; Tsuzuki and Guo, 2004; Usher and McClelland, 2004; Tsuzuki and Busemeyer, 2012). According to the regularity principle, adding a third alternative to a core choice set (two alternatives) does not increase the probability of choosing either of the two original alternatives (Huber et al., 1982). A three-alternative decision-making task wherein each alternative has two attributes (e.g., the quality and price of a consumer product) is often used in studies of the attraction effect. Two of the three alternatives (target and competitor) form a trade-off, such that one is better than the other in one attribute, but worse in the other. The average choice proportions for the target versus the competitor are set as equal based on preliminary surveys. When the third alternative (a decoy) is slightly inferior to the target in one or both attributes, then the probability of choosing the target, rather than the competitor, increases. This bias in decision making is termed the attraction effect (Huber et al., 1982; Hedgcock and Rao, 2009; Huber et al., 2014). This effect warrants special attention because it reflects the context-dependent violation of axioms (e.g., the regularity principle) that are considered fundamental to rational choice.

Numerous explanations have been provided for decoy effects, such as the attraction, compromise, and similarity effect. Several dynamic computational models have been proposed, particularly the multi-alternative decision field theory (MDFT; Roe et al., 2001) and the leaky competing accumulator (LCA) model (Usher and McClelland, 2004). Both models attempt to integrate several mechanisms into a single computational framework. Although MDFT and LCA models differ in several ways, including how units of connectionist networks influence the integration of preferences for alternatives, both models assume this influence to involve a series of stochastic shifts in attention across time, rather than a static process of evaluation. In both models, participants are assumed to search the choice set by means of frequent comparisons of the alternatives, one attribute at a time.

While the authors who proposed the MDFT and LCA models seemed to assume the importance of explaining different decoy effects using the single complex framework, the explanation of these effects based on the dual-process theory has also become widely accepted (Wedell, 2015). Many psychologists have explored the difference between fast and slow thinking over the last 30 years, and this difference has been called the dual process theory (Sloman, 1996; Stanovich and West, 2000; Kahneman, 2003, 2011; Wedell, 2015). These two contradictory aspects of human cognition have led researchers to postulate two thought systems, described as System 1 and System 2 thinking. System 1 thinking is characterized as quick, effortless, automatic, parallel, perceptual, or intuitive, with no sense of voluntary control. System 2 is characterized as slow, effortful, controlled, serial, or reasoned, and is often associated with the subjective experience of choice and concentration. Kahneman (2011) summarized that most of what people think and do originates in System 1, but when they run into difficulty, System 2 supports more detailed and specific processing that may solve the current problem.

Presupposing the dual-process theory (Wedell, 2015), dynamic models like MDFT and LCA (sequential sampling models) are considered to be built upon System 1 processing (but can simulate System 2 processes using each unique mechanism). Previous experiments have indicated that the attraction effect reflects the operation of System 1 rather than System 2. As an example of the System 1-based explanation of the attraction effect, according to MDFT, comparisons of the dominated decoy with the other two alternatives produces a negative preference state for the decoy. Then this negative activation from the decoy feeds through negative inhibitory connections (distance-dependent lateral inhibition) to the closely positioned target (dominant alternative) and the far positioned competitor. As the multiplication of two negative values produces positive activation, the decoy sends positive activation to the other two alternatives. The effect of this activation on a similar alternative (target) is greater than the dissimilar alternative (competitor) because of the strength of the distance-dependent lateral inhibition. Therefore, the target receives more bolstering than the competitor, accounting for the attraction effect (Roe et al., 2001; Usher and McClelland, 2004).

As the attraction effect is assumed to be a result from using automatic System 1 processes and demands low cognitive resources, this effect should increase when the allocation of cognitive resources is prevented. In contrast, decisions based on System 2 (controlled, demanding more cognitive effort) would decrease this effect. For instance, Pocheptsova et al. (2009) demonstrated an increase in the attraction effect when a multi-alternative decision-making task was preceded by a Stroop task or a self-regulation task, which are considered to deplete cognitive resources (i.e., the relative contribution of System 1 becomes greater than that of System 2). Furthermore, Masicampo and Baumeister (2008) reported a decrease in the attraction effect when participants ingested sugar and had an increased blood glucose level, which is believed to increase cognitive resources. These results support the notion that effortful processing, which requires more cognitive resources and involves System 2, disrupts the attraction effect. More recently, using a dual-task paradigm, Tsuzuki et al. (2018) demonstrated the influence of cognitive-resource allocation on the attraction effect when participants were performing a visual multi-alternative choice task. In this experiment, participants simultaneously executed a visual choice task and an auditory probe task, which was irrelevant to the visual task in itself. The attraction effect was significantly greater when participants performed an auditory oddball task (i.e., detecting a target that rarely appeared in an oddball sequence) concurrently with a visual choice task than when they ignored auditory stimuli. This finding indicates that whether or not resources are allocated, at the precise moment, to the visual decision making is directly related to the occurrence of the attraction effect.

Although a relationship between the amount of cognitive resources and magnitude of the attraction effect has been suggested, the source of the disruption that accompanies resource depletion remains unclear. Because previous studies have manipulated the total pool of cognitive resources (Masicampo and Baumeister, 2008; Pocheptsova et al., 2009) or the allocation of cognitive resource in each trial (Tsuzuki et al., 2018), they could not examine the time course of cognitive-resource allocation affecting the occurrence of the attraction effect. Previous studies that used eye-movement monitoring as a process-tracing method during decision making proposed that the decision processes that occur prior to the response could be divided into three stages (Russo and Leclerc, 1994; Glaholt and Reingold, 2011; Noguchi and Stewart, 2014). The first stage involves an initial screening process, in which alternatives are processed selectively based on their relevance. The second stage is linked to evaluation and comparison processing, wherein competitive alternatives are compared and the majority of the decision making occurs. The final segment of the decision course involves validation of the alternatives immediately prior to making the final choice. To understand the relationship between cognitive resources and the attraction effect better, it is necessary to examine the time course of cognitive-resource allocation in a decision-making task in more detail.

To evaluate the amount of cognitive resources that are allocated to each alternative in each of the abovementioned stages, we here used a task-irrelevant probe technique, wherein task-irrelevant auditory probes (that should be ignored) were presented to participants as they viewed a series of alternatives, and measured participants’ electroencephalographic (EEG) responses to the probes. The task-irrelevant probe technique assumes that cognitive resources are capacity-limited, and that, therefore, residual cognitive resources that can be used for processing of task-irrelevant auditory probes are reduced if participants allocate more cognitive resources to the main visual task. This assumption predicts that the amplitudes of event-related potentials (ERPs) elicited by the auditory probes should *decrease* with *increasing* allocation of cognitive resources to the visual task, because the cognitive resources that can be used for the processing of auditory probes are reduced.

Previous studies have shown that the amplitudes of ERPs elicited by ignored auditory probes decrease when more cognitive resources are allocated to visual information (Kramer et al., 1995; Ullsperger et al., 2001; Allison and Polich, 2008; Miller et al., 2011; Takeda and Kimura, 2014; Takeda et al., 2014). For example, Takeda and Kimura (2014) demonstrated that the amplitude of the N1 component of ERPs elicited by task-irrelevant auditory probes decreases when participants watch an interesting video clip versus a boring video clip, because the interesting video clip requires more cognitive resources than the boring video clip. The N1 component is considered to be associated with stimulus filtering and involuntary attention shifting (Escera et al., 1998). A previous study has suggested that the N1 component elicited by task-irrelevant probes is related to the amount of cognitive resources allocated to a visual task in a passive manner, whereas the later P2 component reflects those allocated to a visual task in an active manner (Takeda et al., 2016). Because the participants passively viewed alternatives in a three-alternative decision-making task, we estimated the amplitudes of the N1 components elicited by task-irrelevant auditory probes. Based on previous findings, we expected that if the participants were focused on the visual information during the task by using System 2, then they would allocate more cognitive resources to it; thus, the N1 amplitude elicited by the auditory probes would decrease.

It should be noted that, because the N1 component is considered to mainly reflect sensory rather than cognitive processes (e.g., Kok, 1997), one may think that the variations of the N1 amplitude correspond to the processing in System 1 and those of later components correspond to System 2. However, this is not the case in the task-irrelevant probe technique; that is, the N1 amplitudes elicited by task-irrelevant auditory probes can decrease with an increase of task difficulty even when the task requires cognitive efforts (i.e., processing in System 2). Indeed, previous studies using the task-irrelevant probe technique have demonstrated the modulation of N1 amplitude in various effortful cognitive tasks, such as performing a radar-monitoring task (Kramer et al., 1995), playing a shooting video game (Allison and Polich, 2008), playing a falling block puzzle game (Miller et al., 2011), watching a video clip (Takeda and Kimura, 2014), and driving a vehicle (Takeda et al., 2016). Furthermore, Ullsperger et al. (2001) compared the effects of perceptual (gauge monitoring) and cognitive (mental arithmetic) tasks on the N1 component and found that the N1 amplitude decreased when participants performed the mental arithmetic task concurrently with the gauge monitoring task compared to when they performed only the gauge monitoring task. Because the mental arithmetic task plausibly involves System 2 processing, it is reasonable to consider that the amplitude of the N1 component elicited by task-irrelevant probes can reflect the consumption of cognitive resources in System 2. As mentioned, the task-irrelevant probe technique allows us to assess how much cognitive resources consumed in the main task indirectly. Therefore, it is considered that the processing in System 1, which consumes few cognitive resources, has little influence in the ERPs elicited by task-irrelevant probes.

The main goal of the present study was to investigate the relationship between the time course of cognitive-resource allocation (i.e., use of System 2) in a three-alternative decision-making task and the presence/absence of the attraction effect. For this purpose, three alternatives were visually presented in a sequence, and the amount of cognitive resources allocated to the alternatives was estimated using the task-irrelevant auditory probe technique described above. If effortful and elaborative processing (System 2) disrupts the attraction effect, then the amount of cognitive resources allocated to the alternatives in one or more processing stages should be greater when the participant chooses the competitor over the target. Thus, in trials where the participants choose the competitor and in which there should be no attraction effect, task-irrelevant probes should elicit smaller N1 amplitudes than in trials where participants choose the target and in which the attraction effect should occur. The advantage of the task-irrelevant probe technique is that the state of cognitive-resource allocation can be examined with high temporal resolution; that is, the amount of cognitive resources allocated to the series of alternatives can be determined precisely.

Following the methods employed by previous eye-movement studies (Russo and Leclerc, 1994; Glaholt and Reingold, 2011; Noguchi and Stewart, 2014), we performed a time-series analysis of the N1 amplitudes by dividing the data of each trial into three epochs. If the amount of cognitive resources allocated to processing the series of alternatives is critical for the presence or absence of the attraction effect, then differences in the N1 amplitude between those trials in which participants choose the target and those in which they choose the competitor should be observed in one or more epochs. In particular, N1 amplitudes were expected to be larger for trials in which the target was chosen while the converse was expected for trials in which the competitor was chosen (i.e., the N1 amplitude will be smaller relative to the amplitudes associated with target choices).

## Materials and Methods

### Participants

Thirty young adults (mean age = 22.67 years, standard deviation = 2.02; age range = 19–29 years; two women) participated in this study. All participants reported normal or corrected-to-normal vision and normal hearing. They received payment for their participation. The study was approved by the safety and ethics committees of Rikkyo University and the National Institute of Advanced Industrial Science and Technology, and was conducted after each of the participants had provided written informed consent. They were recruited from a participant pool without any restriction.

### Apparatus and Stimuli

The visual stimuli were presented on a 17-inch cathode-ray-tube display (Trinitron Multiscan G220; Sony, Tokyo, Japan), and the auditory stimuli were presented binaurally via headphones (HD265; Sennheiser, Wedemark, Germany). The ERPs were measured using a Neurofax EEG-1200 system (Nihon Kohden, Tokyo, Japan). Both visual and auditory stimuli were controlled by the same computer operating Mac OSX, MATLAB (Mathworks Inc., Natick, MA, United States), and the Psychophysics Toolbox (Brainard, 1997; Pelli, 1997).

As in previous studies (Pettibone and Wedell, 2000; Tsuzuki and Busemeyer, 2012), we conducted preliminary surveys to determine the inherent value of each attribute and subsequently developed 24 choice sets. Furthermore, by slightly increasing or decreasing the two attribute values of one core alternative and computing a new attribute value for the other core alternative based on a regression equation, we doubled the number of choice sets (48; see the Supplementary Material). Each choice set contained two core alternatives (target and competitor) and a third alternative (decoy), based on a single type of consumer product or service, all described by two attributes (e.g., quality, functional capability, design, and price). Across the 48 choice sets, the average choice proportions for the target versus the competitor were not significantly different in preliminary surveys. The decoy was created by lowering the values of both target attributes by one-sixth of the difference between the core alternatives. As shown in the Supplementary Material, “A” was a target when “A,” “B,” and “D_A_” were presented as alternatives, whereas “B” was a target when “A,” “B,” and “D_B_” were presented as alternatives (within-subjects manipulation).

For each alternative, the name of the product or service, the two attributes, and their values were presented in a bulleted list written in white Japanese characters against a gray background. At a viewing distance of approximately 60 cm, each visual stimulus was surrounded by a colored rectangle (red, green, or blue), which subtended a horizontal visual angle of 9.5° and a vertical visual angle of 5.7°.

Twelve pure tones were used as auditory stimuli. The stimuli had frequencies of 500, 600, 700, 800, 900, 1000, 1100, 1200, 1300, 1400, 1500, and 1600 Hz, and were presented in random order. The duration of each stimulus was 50 ms, including 10-ms rise and fall times, and the stimulus-onset asynchrony varied from 400 to 800 ms (mean = 600 ms). The sound pressure level was approximately 75 dB. Notably, varying frequencies of tones within a sequence can reduce the neural refractory effect and increase the sensitivity of the N1 amplitude to the allocation of cognitive resources (see Takeda and Kimura, 2014).

### Procedure

**FIGURE 1:**
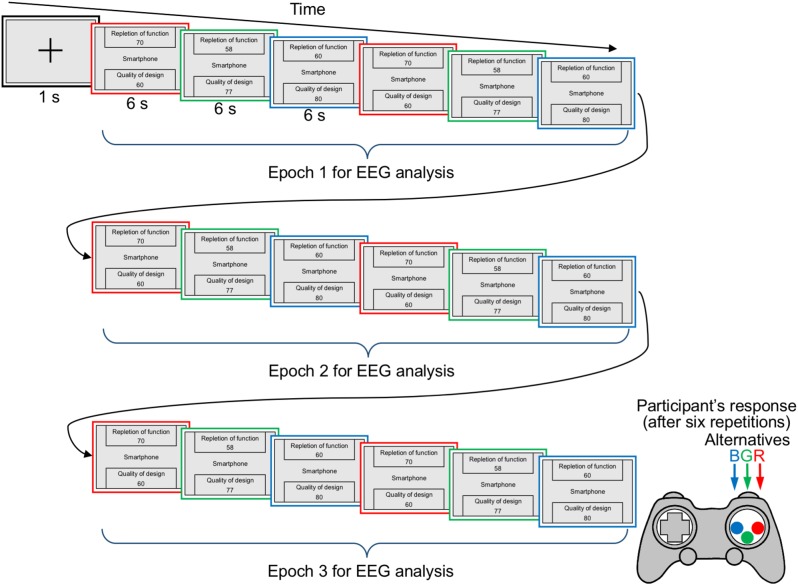
Time course of a single trial in the experiment. Each alternative was displayed for 6 s with a 1-s inter-stimulus interval. The three alternatives were presented repeatedly six times for each choice set. Later, an evaluation screen appeared and remained until the participant responded. The auditory probes were successively presented binaurally via headphones as the participants viewed the alternatives. The participants’ EEG responses were measured during the whole trial and were averaged separately based on the three time-series epochs (each epoch included six stimuli: 3 alternatives × 2 repetitions).

Each participant performed 48 trials, one for each choice set. Each trial began with the presentation of a fixation point for 1 s, followed by the sequential presentation of the alternatives (target, competitor, and decoy). Each alternative was displayed for 6 s with a 1-s inter-stimulus interval. The three alternatives were repeatedly presented six times for each choice set (i.e., 18 stimuli were presented in total: see Figure 1). There were six permutations of the presentation order of the target, competitor, and decoy. We strictly controlled the frequencies of these six presentation orders so that they were equally counterbalanced (however, in each trial, the order of the three types of alternatives was the same in six repetitions). Additionally, each of the rectangles surrounding the three alternatives was randomly assigned one of three colors (red, green, or blue).

After the alternatives were repeatedly presented six times for each choice set, the participants were required to choose one alternative that they desired to purchase by pressing the appropriate button on a gamepad. The next trial began after the participant’s response. The presentation order of the 48 choice sets was randomized across the participants. As explained above, the auditory probes were successively presented binaurally via headphones as the participants viewed the alternatives. The participants were provided with a short break after every six trials.

Participants were instructed that they would view a series of three alternative choice sets (48 consumer products) repeatedly presented several times, for which surrounded colored rectangles are assigned one of the three colors (red, green, or blue). Participants would need to decide which product to buy in each set at the end of repeated presentation using a gamepad corresponding to the frame color of the alternative. They were also informed that the three alternatives of each choice set differed in two features and the other features of each choice set are equivalent. In addition, they were instructed to ignore the auditory stimuli (i.e., the task-irrelevant probes).

### Recordings

The participants’ EEG responses were measured using a digital amplifier (Neurofax EEG1200; Nihon-Kohden), and silver-silver chloride electrodes were placed at 27 scalp sites (Fp1, Fp2, F7, F3, Fz, F4, F8, FCz, T3, C3, Cz, C4, T4, T5, CPz, P3, Pz, P4, T6, PO7, PO8, O1, Oz, O2, O9, Iz, and O10, according to the extended International 10–20 System), with AFz as the ground electrode. To monitor eye movements, a vertical electrooculogram (EOG) was recorded using electrodes placed above and below the right eye, and a horizontal EOG was recorded from the outer left and right canthi. The impedances of all electrodes were kept below 10 kΩ. The EEG and EOG signals were digitized at a sampling rate of 1000 Hz, and the time constant was set to 5 s. The EEG signals were re-referenced to mathematically averaged earlobes (A1-A2) off-line. The EEG and EOG signals were band-pass filtered at 0.1–30 Hz using a second-order Butterworth filter.

### Data Analysis

The proportions of the choices of the target, competitor, and decoy were calculated for each participant. The proportions of the choices of the target, competitor, and decoy were subjected to a repeated measure analysis of variance (ANOVA).

To compute the ERPs elicited by the auditory probes, the data window of the EEG signals was set from –100 to 500 ms relative to probe onset. To remove eye-blink-related artifacts, an independent component analysis, as implemented in EEGLAB version 11.0.4.3 (Delorme and Makeig, 2004), was adopted. In the averaging procedure, the data containing signal changes that exceeded ±80 μV at any of the EEG sites (8.6% of epochs on average) were excluded. The EEG signals were averaged separately based on the three time-series epochs. That is, the presentation period (including 3 alternatives × 6 repetitions) were divided into three time-series epochs (including 3 alternatives × 2 repetitions), which are considered to correspond to the stages of the initial screening processes, evaluation and comparison processes, and validation processes (e.g., Glaholt and Reingold, 2011). Because it is plausible that the attraction effect’s occurrence depends mainly on the evaluation and comparison processes (Russo and Leclerc, 1994), we are especially interested in the second epoch. Additionally, EEG signals were averaged separately based on the participants’ choices (trials in which participants chose the target or trials in which they chose the competitor). Note that the number of trials in which the participants chose the decoy was too small to compute ERPs, and these trials were therefore not included in the ERP analysis.

Event-related potentials were evaluated relative to a 100-ms pre-stimulus baseline. Since frontocentral negativity (i.e., the N1 component) was observed, and its peak latency at FCz was 92 ms when ERPs were collapsed across the six types, the mean amplitude of the N1 component was computed within the 80–104-ms (i.e., 92 ± 12-ms) latency window at FCz following the methods used by Takeda and Kimura (2014). The mean amplitudes of the N1 component were subjected to a two-way ANOVA with the three epochs and the participant’s choice (target or competitor) as within-participant variables.

## Results

### Proportions of Choices

**FIGURE 2:**
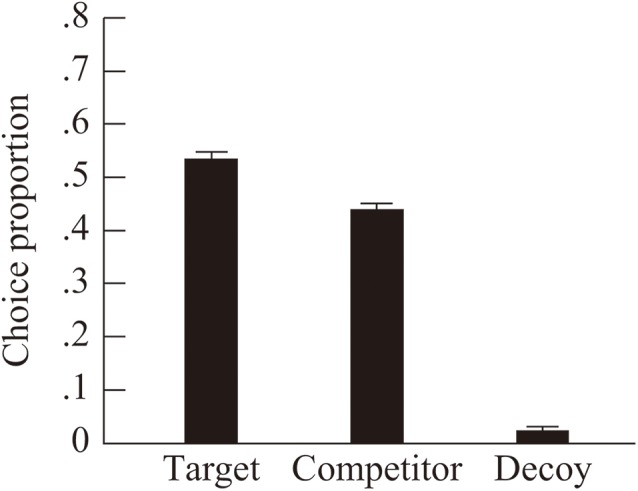
Mean choice proportions for the target, competitor, and decoy. Error bars indicate the standard error of the mean.

The average choice proportions (*SE*) of the target, competitor, and decoy were 0.54 (0.01), 0.44 (0.01), and 0.02 (0.01), respectively (see Figure 2). A one-way ANOVA (three levels: target, competitor, and decoy) revealed a significant main effect, *F*(2, 58) = 507.73, *p* < 0.001, ${\text{η}}_{\text{p}}^{2}$ = 0.95. A *post hoc* analysis (with Bonferroni correction) revealed that the choice proportion of the target was significantly higher than the proportions of the competitor and decoy (*p* < 0.001 for both comparisons). Further, the choice proportion of the competitor was significantly higher than that of the decoy (*p* < 0.001).

### ERPs

**FIGURE 3:**
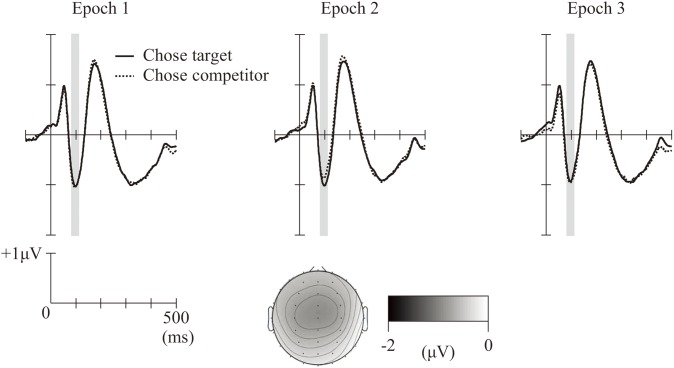
Grand-averaged event-related potential (ERP) waves at FCz for the three epochs. Solid lines represent ERPs during trials in which the participants chose the target, and dashed lines represent ERPs during trials in which the participants chose the competitor. Gray bars indicate the N1 range (92 ± 12 ms). A topographical map illustrates the mean amplitudes in the N1 range (collapsed across trials).

**FIGURE 4:**
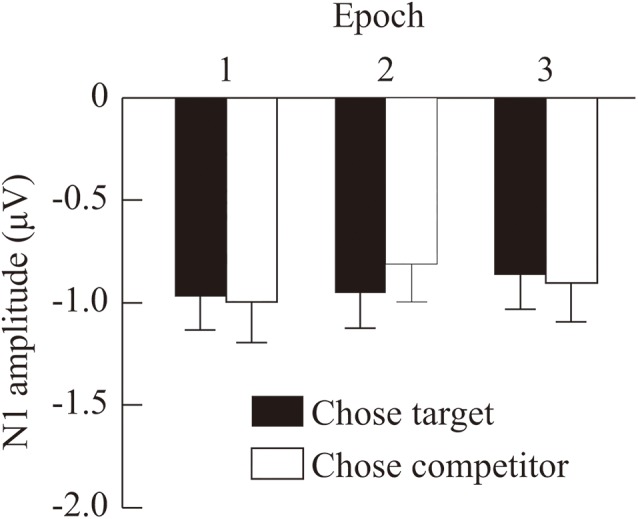
Mean N1 amplitudes in the three epochs. Black bars represent the mean N1 amplitudes during trials in which the participants chose the target, and white bars represent the mean N1 amplitudes during trials in which the participants chose the competitor. Error bars indicate the standard error of the mean.

The grand-averaged ERP waves at FCz and the topographical map representing the mean amplitudes of the N1 range are shown in Figure 3. The mean number of averaged epochs (*SE*) of Epoch1-chose-target (i.e., the first two repetitions of the choice set within the trial in which the target was chosen, regardless of its position in the three-alternative sequence), Epoch1-chose-competitor, Epoch2-chose-target, Epoch2-chose-competitor, Epoch3-chose-target, and Epoch3-chose-competitor were 1674.7 (39.1), 1377.0 (34.8), 1683.7 (37.8), 1380.8 (34.7), 1679.6 (38.2), and 1381.5 (35.4), respectively. Averaging more than one thousand epochs allows us to examine the amplitudes of the N1 component with a high signal/noise ratio (Luck, 2005). All ERP waves had a frontocentral negative component at around 92 ms, which was consistent with the N1 component. The mean amplitudes of the N1 component (*SE*) of Epoch1-chose-target, Epoch1-chose-competitor, Epoch2-chose-target, Epoch2-chose-competitor, Epoch3-chose-target, and Epoch3-chose-competitor were –0.97 (0.16), –1.00 (0.19), –0.95 (0.17), –0.81 (0.18), –0.86 (0.16), and –0.90 (0.19) (see Figure 4). An ANOVA of the mean amplitudes indicated a significant interaction between the epochs (first, second, or third) and the participant’s choice (target or competitor), *F*(2, 58) = 3.39, *p* = 0.041, ${\text{η}}_{\text{p}}^{2}$ = 0.11. A *post hoc* analysis (with Bonferroni correction) revealed that, in the second epoch, the mean N1 amplitude was significantly smaller for trials in which the participants chose the competitor than for trials in which they chose the target (*p* = 0.041). Furthermore, the mean N1 amplitude for trials in which participants chose the competitor was significantly smaller in the second epoch than in the first epoch (*p* = 0.009).

## Discussion

In the present study, participants were more likely to choose the target rather than the competitor during a multi-alternative decision-making task, indicating the presence of an attraction effect. The electrophysiological results demonstrate that, in the second epoch (i.e., during the 3rd and 4th repetitions of alternatives), the mean amplitude of the N1 component when viewing the alternatives was smaller when the participants chose the competitor (i.e., trials with no attraction effect) than when they chose the target (i.e., trials with a possible attraction effect). These results are consistent with previous empirical evidence suggesting that effortful processing (the allocation of more cognitive resources to the alternatives) disrupts the attraction effect in multi-alternative decision-making tasks, and are consistent with the theoretical framework of context-dependent choice based on Systems 1 and 2 of the dual-process theory (Masicampo and Baumeister, 2008; Pocheptsova et al., 2009).

As explained in the introduction, theoretically, the mental processes that occur prior to the final choice can be divided into three stages: (1) initial screening processes, (2) evaluation and comparison processes, and (3) validation of the alternatives immediately prior to the final choice (Russo and Leclerc, 1994; Glaholt and Reingold, 2011; Noguchi and Stewart, 2014). Russo and Leclerc (1994) argued that the bulk of the deliberation and evaluation takes place in the second stage, particularly that of weighing alternatives that are being seriously considered. In this study, we performed a time-series analysis of N1 amplitudes by dividing the data of each trial into three epochs. It is very likely that the N1 amplitudes indicate that the allocation of cognitive resources to alternatives in the second epoch is strongly related to the presence or absence of the attraction effect. Although these three stages could be assumed to represent the average flow of processing in our experimental task, the possibility that some individual differences among participants exist in the decision-making stages cannot be ruled out. Furthermore, although it is likely that the second stage reflects the evaluation and comparison processing, it was not possible to conclude which type of resource allocation, related to which type of pair-wise comparison of alternatives, is important (or not important) for the occurrence of the attraction effect. Further, as a more fundamental problem, it is difficult to say that our experimental results present sufficient evidence to support the association of N1 in the second epoch with the evaluation/comparison stage. It is important to undertake more research to gather further evidence and substantiate this association.

Malaviya and Sivakumar (2002) reported that cognitive resource allocation for the processing of product information (descriptions of two attributes of the options) as well as for choice justification (participants’ explanations of the rationale for their preferences) affects the occurrence of the attraction effect. The present study focused on the allocation of cognitive resources to product information but not to choice justification. In a realistic purchase setting, because consumers sometimes justify their choices, it is necessary to consider resource allocation to choice justification to fully understand the properties of the attraction effect. This is related to the importance of considering external validity, which refers to the possibility of generalizing an observed causal relationship to and across different measures, persons, settings, and times (Calder et al., 1982). Lichters et al. (2015) recommended seven guidelines from the perspective of realistic consumer research to ensure external validity for implementing context-effect experiments. Laboratory setting studies have not sufficiently adhered to some of these guidelines. Future research should therefore systematically examine these issues.

By using an electrophysiological index that can directly measure amounts of cognitive resources with high temporal resolution, that is, EEG, the results of the present study suggest that the allocation of greater cognitive resources during the evaluation and comparison processes, but not during the initial screening and final validation processes, disrupts the attraction effect. To our knowledge, the present study is the first to provide objective evidence that the attraction effect is based on non-deliberative evaluation and comparison processes. Our findings highlight the importance of the evaluation and comparison processes, and are consistent with the findings of recent eye-movement studies that demonstrate that the pattern of comparison among alternatives can predict the magnitude of the attraction effect (Chiba and Tsuzuki, 2014; Noguchi and Stewart, 2014). Considering the present findings together with the notion that the attraction effect is a consequence of relatively effortless and intuitive processing (System 1; Masicampo and Baumeister, 2008; Pocheptsova et al., 2009), it is reasonable to think that the comparison among alternatives is executed by System 1 rather than by System 2, in order to enhance the preference for the target.

As mentioned in the introduction, many theories and models have been presented to explain the different types of decoy effects, including the attraction effect (see, e.g., Busemeyer et al., 2018; Noguchi and Stewart, 2018; Turner et al., 2018, for recent reviews). Soltani et al. (2012) have proposed a biophysical computational model to explain the attraction effect based on adjustments of neural representations according to the sets of option attributes. In contrast, to account for the attraction effect in psychological models, such as the context-dependent advantage (CAD) model (Tversky and Simonson, 1993), MDFT, and LCA, each core mechanism involves the comparison between each pair of alternative attributes. As the core mechanism of these models can be considered as included in System 1 (Wedell, 2015), we firmly believe that our experimental results are compatible with the assumptions of these major models of the decoy effects.

In summary, we investigated the relationship between the allocation of cognitive resources and the robust attraction effect (i.e., context-dependent bias) in multi-alternative decision making. To do this, we used a novel experimental paradigm wherein a task-irrelevant auditory probe was used during the sequential presentation of choice alternatives. We found that the occurrence of the attraction effect depended on the allocation of cognitive resources during the processing of alternatives. Specifically, the electrophysiological results indicate that the allocation of more cognitive resources to the alternatives in the second epoch (considered to represent the evaluation and comparison stage) disrupted the attraction effect. This result is consistent with the assumption that effortless and intuitive comparisons between alternatives are critical for the occurrence of the attraction effect.

## Ethics Statement

This study was approved by the safety and ethics committees of Rikkyo University and the National Institute of Advanced Industrial Science and Technology, and it was conducted after each participant gave their written informed consent in accordance with the Declaration of Helsinki.

## Author Contributions

TT and YT designed the experiments. IC and YT performed the experiments and analyzed the data. YT drew the figures. All authors wrote and revised the manuscript and approved the final version of the manuscript to be published.

## Conflict of Interest Statement

The authors declare that the research was conducted in the absence of any commercial or financial relationships that could be construed as a potential conflict of interest.
