# Supplementary material for: Effortful Processing Reduces the Attraction Effect in Multi-Alternative Decision Making: An Electrophysiological Study Using a Task-Irrelevant Probe Technique
Source: Front Psychol. 2019 Apr 24;10:896. doi: 10.3389/fpsyg.2019.00896 (PMC6491867; doi:10.3389/fpsyg.2019.00896)
Supplement: Supplementary file 1 [file Table_1.pdf]

*Supplementary Material*

# Effortful Processing Reduces the Attraction Effect in Multi-alternative Decision Making: An Electrophysiological Study Using a Task-irrelevant Probe Technique

**Takashi Tsuzuki*, Yuji Takeda, and Itsuki Chiba**

*** Correspondence:** Takashi Tsuzuki: tsuzuki@rikkyo.ac.jp

Table 1 | The trinity choice sets used in the experiment.

| Consumer product or service | Two attributes | Set 1 | | | | Set 2 | | | |
|---|---|---|---|---|---|---|---|---|---|
| | | A | B | $D_A$ | $D_B$ | A | B | $D_A$ | $D_B$ |
| Athletic shoes | Quality of design (1–100) | 80 | 65 | 78 | 63 | 77 | 62 | 75 | 60 |
| | Comfort in wearing (1–100) | 65 | 75 | 63 | 73 | 67 | 77 | 65 | 75 |
| School bag | Weight (Kg) | 0.80 | 1.20 | 0.87 | 1.27 | 0.70 | 1.10 | 0.77 | 1.17 |
| | Quality of design (1–100) | 65 | 80 | 63 | 78 | 63 | 78 | 61 | 76 |
| Traveler's bag | Quality of design (1–100) | 80 | 65 | 78 | 63 | 78 | 63 | 76 | 61 |
| | Weight (Kg) | 1.20 | 0.80 | 1.27 | 0.87 | 1.10 | 0.70 | 1.17 | 0.77 |
| Coat | Quality of design (1–100) | 70 | 80 | 68 | 78 | 68 | 78 | 66 | 76 |
| | Price (thousand Yen) | 7.0 | 10.0 | 7.5 | 10.5 | 6.4 | 9.4 | 6.9 | 9.9 |
| Exercise clothes | Price (thousand Yen) | 10.0 | 7.0 | 10.5 | 7.5 | 9.4 | 6.4 | 9.9 | 6.9 |
| | Quality of design (1–100) | 80 | 70 | 78 | 68 | 78 | 68 | 76 | 66 |
| Wristwatch | Quality of design (1–100) | 80 | 70 | 78 | 68 | 82 | 72 | 80 | 70 |
| | Price (thousand Yen) | 9.00 | 5.00 | 9.67 | 5.67 | 9.80 | 5.80 | 10.47 | 6.47 |
| MP3 Player | Price (thousand Yen) | 12.00 | 8.00 | 12.67 | 8.67 | 10.70 | 6.70 | 11.37 | 7.37 |
| | Sound quality (1–100) | 76 | 70 | 75 | 69 | 74 | 68 | 73 | 67 |
| Earphones | Sound quality (1–100) | 84 | 70 | 82 | 68 | 82 | 68 | 80 | 66 |
| | Price (thousand Yen) | 4.00 | 3.00 | 4.17 | 3.17 | 3.80 | 2.80 | 3.97 | 2.97 |
| Electronic dictionary | Repletion of dictionaries (1-100) | 75 | 65 | 73 | 63 | 77 | 67 | 75 | 65 |
| | Price (thousand Yen) | 20.0 | 15.0 | 20.8 | 15.8 | 21.0 | 16.0 | 21.8 | 16.8 |
| Digital camera | Image quality (1-100) | 70 | 76 | 69 | 75 | 72 | 78 | 71 | 77 |
| | Repletion of functions (1-100) | 80 | 65 | 78 | 63 | 75 | 60 | 73 | 58 |
| Smartphone | Repletion of functions (1-100) | 60 | 70 | 58 | 68 | 59 | 69 | 57 | 67 |

| | | | | | | | | | |
|---|---|---|---|---|---|---|---|---|---|
| | Quality of design (1–100) | 80 | 60 | 77 | 57 | 82 | 62 | 79 | 59 |
| Notebook computer | Repletion of functions (1-100) | 65 | 75 | 63 | 73 | 67 | 77 | 65 | 75 |
| | Price (thousand Yen) | 60 | 90 | 65 | 95 | 66 | 96 | 71 | 101 |
| Tablet personal computer | Price (thousand Yen) | 15.0 | 20.0 | 15.8 | 20.8 | 16.0 | 21.0 | 16.8 | 21.8 |
| | Repletion of functions (1-100) | 65 | 75 | 63 | 73 | 67 | 77 | 61 | 71 |
| Electronic keyboard | Number of distinctive functions | 13 | 7 | 12 | 6 | 14 | 8 | 13 | 7 |
| | Sound quality (1–100) | 70 | 80 | 68 | 78 | 68 | 78 | 66 | 76 |
| Liquid crystal display television | Screen size (inch) | 19 | 26 | 18 | 25 | 20 | 27 | 19 | 26 |
| | Image quality (1-100) | 80 | 70 | 78 | 68 | 78 | 68 | 76 | 66 |
| Single sofa | Price (thousand Yen) | 14.00 | 10.00 | 14.67 | 10.67 | 13.20 | 9.20 | 13.87 | 9.87 |
| | Comfort in seating (1–100) | 75 | 65 | 73 | 63 | 73 | 63 | 71 | 61 |
| Office chair | Comfort in seating (1–100) | 65 | 75 | 63 | 73 | 63 | 73 | 61 | 71 |
| | Price (thousand Yen) | 10.00 | 14.00 | 10.67 | 14.67 | 9.20 | 13.20 | 9.87 | 13.87 |
| Bed | Quality of design (1–100) | 60 | 79 | 57 | 76 | 62 | 81 | 59 | 78 |
| | Price (thousand Yen) | 17.0 | 24.0 | 18.2 | 25.2 | 17.7 | 24.7 | 18.9 | 25.9 |
| Bicycle | Price (thousand Yen) | 20.0 | 15.0 | 20.8 | 15.8 | 21.0 | 16.0 | 21.8 | 16.8 |
| | Quality of design (1–100) | 70 | 60 | 68 | 58 | 72 | 62 | 70 | 60 |
| Gas scooter | Quality of design (1–100) | 75 | 60 | 73 | 58 | 77 | 62 | 75 | 60 |
| | Gas mileage (km per liter) | 60 | 70 | 58 | 68 | 59 | 69 | 67 | 67 |
| Hair salon | Magazine's rating of skill (1–100) | 80 | 70 | 78 | 68 | 78 | 68 | 76 | 66 |
| | Time taken to reach there from home (min) | 37 | 19 | 40 | 22 | 33 | 15 | 36 | 18 |
| Fitness club | Repletion of equipment (1–100) | 80 | 65 | 78 | 63 | 78 | 63 | 76 | 61 |
| | Time taken to reach the club from home (min) | 28 | 15 | 30 | 17 | 26 | 13 | 28 | 15 |
| Rental apartment | Monthly rent (thousand Yen) | 55.0 | 60.0 | 55.8 | 60.8 | 53.9 | 58.9 | 54.7 | 59.7 |
| | Walking distance from the station to the apartment (min) | 19 | 10 | 21 | 12 | 21 | 12 | 23 | 14 |
| Restaurant | Magazine's rating of skill (1–100) | 75 | 65 | 73 | 63 | 73 | 63 | 71 | 61 |
| | Time taken to reach the restaurant from school (min) | 19 | 10 | 20 | 11 | 17 | 8 | 18 | 9 |

Note: Choice sets 1 and 2 consisted of 24 consumer products or services, their two attributes, and three alternatives (the target, competitor, and decoy), which also had two attribute values. “A” was a target when “A,” “B,” and “$D_A$” were presented as alternatives, whereas “B” was a target when “A,” “B,” and “$D_B$” were presented as alternatives.
